# Exploring reasons for attrition among vulnerable and under-served sub-groups across an online integrated healthy lifestyles service during COVID-19

**DOI:** 10.1177/20503121211054362

**Published:** 2021-10-22

**Authors:** George J Sanders, Carlton Cooke, Paul Gately

**Affiliations:** Carnegie School of Sport, Leeds Beckett University, Leeds, UK

**Keywords:** Attrition, COVID-19, community behavior change, digital interventions, health promotion, health literacy

## Abstract

**Objectives:**

Reported health behaviour change intervention attrition rates vary considerably, from 10% to more than 80%, depending on the type and setting of the treatment programme. A better understanding of the determinants of programme adherence is required. Between March and August 2020, a convenience sample of 44 individual telephone interviews, as well as 42 online Qualtrics surveys took place. The objective was to explore perceived barriers, facilitators, and opportunities for participation, sustained participation as well as initial non-participation to better understand reasons for attrition in online delivery during the COVID-19 lockdown among vulnerable and under-served groups within an Integrated Healthy Lifestyle Service (IHLS).

**Methods:**

A convenience sample of 44 individual telephone interviews, as well as 42 online Qualtrics surveys resulted in a total of 86 (33 male) individuals comprising intervention clients. Clients included children and young people (n = 16), manual workers (n = 7), Black, Asian or Minority Ethnic (n = 19), physical disability (n = 8), learning disability (n = 6), and those from areas of high deprivation (n = 19), as well as Integrated Healthy Lifestyle Service practitioners (n = 11).

**Results:**

The study revealed that more resources and support are needed for Black, Asian or Minority Ethnic; manual worker; learning disability; and high-deprivation sub-groups in order to reduce attrition rates. Specifically, a lack of technological equipment and competence of using such equipment was identified as key barriers to initial and sustained attendance among these vulnerable and under-served sub-groups during the COVID-19 lockdown.

**Conclusion:**

The pattern of differences in attrition during the COVID-19 lockdown suggests that further research is required to explore how best to ensure online health behaviour change offers are scalable and accessible to all.

## Introduction

On 30 January 2020, the pandemic spread of COVID-19 was declared a Public Health Emergency of International Concern by the World Health Organization (WHO).^1^ As of June 2021, the virus has infected more than 175 million people worldwide, causing more than 3,700,000 deaths^1^ and hence, governments across the globe imposed varying degrees of social distancing advice and nationwide lockdowns. Alarmingly, the United Kingdom currently records the seventh highest number of infections (over 4,500,00), and the fifth highest number of deaths (over 128,000) globally.^1^ The tradeoff between protection from COVID-19 and increased risk of inactivity and exposure to energy-dense foods presents already vulnerable populations with a potential ‘no-win’ situation. For instance, where the consequence of protection from acquiring COVID-19 is social isolation and increased inactivity, this could put these same individuals at heightened risk of mental health problems,^2^ chronic diseases, such as cardiovascular disease, stroke,^3^ and increased weight and premature mortality.^4^ In the long term, it is also possible that because of lockdown-associated worsening of underlying health conditions, the associated effects of decreases in physical activity (PA) and increasing food intake during lockdown could actually serve to increase the size of the population that is vulnerable to severe complications from COVID-19 in subsequent epidemic waves. These effects could potentially add additional pressure to the health system during the current or later epidemic waves. However, the COVID-19 pandemic also provides an opportunity for the rollout of novel online behaviour change offers to maintain the delivery of lifestyle interventions remotely. Harnessing the surge in interest, enthusiasm, and acceptance in digitally based behaviour change offers during lockdown has immediately been recognized as an opportunity for service providers.^5^

One of the most efficacious and widely reported strategies for the management of healthy lifestyles are health behaviour change interventions.^6^ However, reported health behaviour change intervention attrition rates vary considerably, from 10% to more than 80%, depending on the type and setting of the treatment programme.^7^ In addition, under-served groups within the population are likely to be under-represented in health behaviour change interventions. Engaging these groups in health promotion is of particular importance as compared to the general population, these groups have more health problems and health care needs.^8^ However, for such programmes to be successful (as measured against a variety of health and lifestyle markers), it is crucial that individuals adhere as best they can to the recommendations provided.^9^

Consequently, this is the first exploratory study to adopt a formative research methodology to inform and refine the design, delivery, and recruitment strategies of an online version of an ongoing county-wide Integrated Healthy Lifestyle Service (IHLS) during the COVID-19 lockdown across the six groups that have shown the highest attrition rates from the past 3 years of delivery of the service pre-COVID-19 during traditional face-to-face modes of delivery. Specifically, these groups are children and young people (CYP) (60% attrition at 6 weeks in the observed IHLS), manual worker (84% attrition at 12 weeks), Black, Asian or Minority Ethnic (BAME) (72% attrition at 12 weeks), physical disability (78% attrition at 12 weeks), learning disability (59% attrition at 12 weeks), and those living in areas of high deprivation (56% attrition at 12 weeks). These sub-groups attended a variety of IHLS sessions including weight management (WM), smoking cessation, and PA interventions.

The objectives of the study are to (1) explore current knowledge and attitudes towards attending online IHLS services during the COVID-19 lockdown; (2) explore perceived barriers, facilitators, and opportunities for participation, sustained participation, as well as initial non-participation, across the attrition groups of completers (>80% session attendance), non-completers (<80% session attendance), and non-attenders during the COVID-19 lockdown. In practice, the IHLS provider can then implement the findings and develop strategies to limit attrition and initial non-participation. Given the purpose and objectives outlined, the Evidence Integration Triangle^10^ was adopted as the overarching theoretical framework to allow for the exploration of the three main evidence-based components of programme/policy, implementation processes, and measures of progress. Hence, results and analysis from this study can then be fed back to the IHLS provider and clients in order to assess, evaluate, and promptly inform adapted, equivalent future involvement. Adoption of such an integrated framework allows for more consistent mapping, evaluation, and incorporation of successful methods and strategies for modifying behavioural determinants.^11^

## Methods

This study provides qualitative data to explore reasons for attrition among vulnerable and under-served groups within a community based IHLS delivered online during the COVID-19 lockdown. The observed IHLS focuses on reducing health inequalities among vulnerable and under-served groups within areas of deprivation. The service is a partnership between a UK-based university and was commissioned by a County Council in the East of England. The UK-based university commits a direct investment into research and evaluation to support the IHLS. Each service is predominantly developed and delivered in line with the required annual key performance indicators (KPIs) as stipulated by the commissioning body.

### Design

A qualitative research design was adopted employing semi-structured interviews and online surveys.

#### Interviews and surveys

Between March and August 2020, a convenience sample of 44 individual telephone interviews, as well as 42 online Qualtrics surveys took place. Convenience sampling has been adopted in interview^12^ and survey^13^ research previously and thus, this study extends the use of these methods. A total of 462 potential service users who were currently attending or had attended one or multiple IHLS services since the transition to online sessions in January 2020 were invited to take part via email, resulting in a 19% acceptance rate. No incentives were provided to potential participants.

Results comprised intervention clients including CYP (n = 16; completers n = 12, non-completers n = 3, non-attenders n = 1), manual workers (n = 7, completers n = 3, non-completers n = 3, non-attenders n = 1), BAME (n = 19; completers n = 8, non-completers n = 9, non-attenders n = 2), physical disability (n = 8; completers n = 3, non-completers n = 5, non-attenders n = 0), learning disability (n = 6; completers n = 0, non-completers n = 6, non-attenders n = 0), and those living in high-deprivation areas (n = 19; completers n = 8, non-completers n = 10, non-attenders n = 1), as well as IHLS practitioners (n = 11). The duration of individual interviews was between 14 and 41 min (mean = 22 min, standard deviation (SD) = 16.4).

The semi-structured discussion guide included eight open-ended questions structured to prompt discussion with probes and follow-up questions adopted as needed (see supplemental material). Interviews were led by a trained facilitator experienced in conducting qualitative data collection methods. Written informed consent was obtained for all participants prior to participation. The online questionnaire was administered via the Qualtrics Online Survey software, with participants providing written informed consent online and completing the same questions as were asked during the interviews. Questions addressed barriers, facilitators, and opportunities towards attending online IHLS sessions during the COVID-19 lockdown. An example question from a section exploring barriers to attendance during lockdown was: ‘Has X (the county-wide IHLS) supported you to keep attending sessions throughout COVID-19? If yes, how? (e.g. virtual sessions, weekly update emails, at home plans etc.) If no, what could have been done better?’ Questions were pilot tested with a selection of research team members and IHLS staff (e.g. team leads and practitioners), with the content and order subsequently agreed upon. Consequently, questions demonstrated aspects of face validity as they were transparent and relevant to the priority population.^14^ Objectivity was maintained by the lead investigator as the resultant qualitative data aligned to the *a priori* Evidence Integration Triangle^10^ framework and was fit to serve as evidence for satisfying the research question.^15^

Institutional ethical approval was received by Leeds Beckett University’s Research Ethics Sub Committee (approval number 68268). All data collection locations were free from background noise, where interviewees could be overlooked but not overheard. Interview data were digitally recorded and transcribed verbatim. The text for each data collection session was sequentially labelled with numbers to identify the sentences that belonged to the participant or interviewer. All data were anonymized and transcripts coded throughout to ensure confidentiality. Verbatim transcripts were read and re-read to allow familiarization with the data. Transcripts were analysed in this manner until data saturation had been achieved and no new information had been obtained.

### Data coding and analysis

The pen profile approach presents findings from content analysis via a diagram of composite key major and minor themes. In summary, deductive content analysis was initially adopted to categorize interview and survey data into an *a priori* major theme of attrition during COVID-19. Inductive analysis then allowed for minor themes to be created beyond the *a priori* major theme via categorisation from line-by-line coding. Data were then organized schematically to assist with interpretation of the themes and verbatim quotations used to expand the *a priori* pen profile, provide context, and verify participant responses. Quotations were labelled by participant number (Pn).

Methodological rigour was demonstrated through a process of inter-rater reliability^16^ whereby coding checks were undertaken for a 20% random sample of all data collected from interview transcripts and survey responses independently by a member of the research team. This involved cross-checking placement of data into the *a priori* COVID-19 pen profile. To ensure transparency, credibility, and trustworthiness of the results, any omissions and discrepancies with coding analysis were then identified and discussed until subsequent agreement on data themes in relation to verbatim extracts was reached. Almost 80% and above agreements are considered acceptable.^16^

## Results

**Figure 1.**
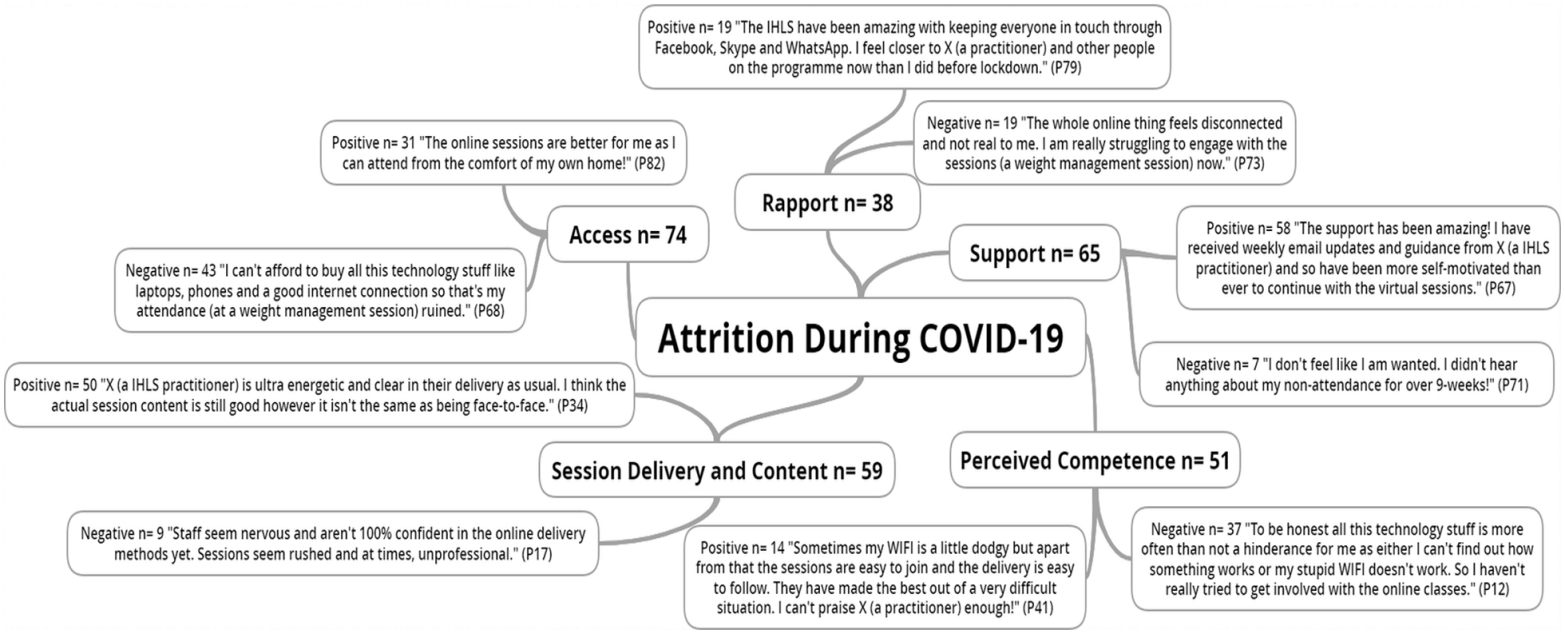
Reasons for attrition among vulnerable and under-served sub-groups across an online integrated healthy lifestyles service during COVID-19. ^*^n = individual mentions per person (multiple mentions not included); Pn = participant number.

The *a priori* attrition during COVID-19 pen profile and emergent themes are presented in Figure 1. Figure 1 outlines largely positive comments in relation to the emergent themes of support, session delivery, and content across clients and practitioners alike.

The support has been amazing! I have received weekly email updates and guidance from X (a IHLS practitioner) and so have been more self-motivated than ever to continue with the virtual sessions. (P67)

The support from other practitioners, my manager and even clients have motivated me most. This online delivery and receipt style are different for all of us, but I genuinely think we have made it work and can continue with this even after this lockdown has ended. We can reach a much wider audience now. The potential for growth across wider communities is huge. (P14)

X (a IHLS practitioner) is ultra-energetic and clear in their delivery as usual. I think the actual session content is still good however it isn’t the same as being face-to-face. (P34)

Mixed comments are noted in relation to the emergent themes of access and rapport.

I can’t afford to buy all this technology stuff like laptops, phones and a good internet connection so that’s my attendance (at a weight management session) ruined. (P68)

X (the IHLS) have been amazing with keeping everyone in touch through Facebook, Skype and WhatsApp. I feel closer to X (a IHLS practitioner) and other people on the programme now than I did before lockdown. (P79)

Largely negative comments are noted in relation to the emergent theme of perceived competence.

I have never been taught how to use a computer, how to setup an email account, how to navigate through online webpages so I wouldn’t know where to start. Since the lockdown I have lost all social connections to the outside world. It is very isolating and daunting. (P28)

Table 1 displays a specific frequency count breakdown of the emergent theme positive and negative mentions across the vulnerable and under-served attrition sub-groups.

**Table 1.** Emergent themes: frequency of positive and negative mentions across vulnerable and under-served sub-groups.

Theme	Attrition sub-group (frequency of positive (+ve) and negative mentions (−ve)						
	CYP (n = 16)	Manual worker (n = 7)	BAME (n = 19)	Physical disability (n = 8)	Learning disability (n = 6)	High deprivation (n = 19)	IHLS practitioners (n = 11)
	+ve: −ve	+ve: −ve	+ve: −ve	+ve: −ve	+ve: −ve	+ve: −ve	+ve: −ve
Access	4:0	7:00	5:14	1:6	6:00	5:15	3:8
Rapport	4:0	1:5	2:2	2:0	1:0	4:12	5:0
Support	7:5	7:0	10:0	8:0	2:0	19:2	5:0
Perceived competence	0:1	3:4	2:16	3:1	1:2	3:13	2:0
Session delivery and content	7:2	5:2	15:1	4:0	2:0	11:4	6:0

CYP: children and young people; BAME: Black, Asian and Minority Ethnic; IHLS; integrated healthy lifestyle service.

## Discussion

This is one of the first studies to adopt an integrated theoretical framework to provide a comprehensive exploration of barriers and facilitators influencing attrition within a UK-based IHLS during the COVID-19 lockdown and the first to focus specifically on vulnerable and under-served sub-groups.

### COVID-19

This study is a first step in exploring both client and practitioner experiences in engaging with online health behaviour change offers during the COVID-19 lockdown. Such findings will be critical in ensuring digital health is used to deliver the best care possible during and beyond the current pandemic, especially to those most at risk of COVID-19-related health issues and health inequalities.

#### Access

We must be aware of the disparities that impact highly deprived and vulnerable individuals and communities, as well as cultural and linguistically diverse communities that may not have access to even basic technology including mobile phones.^17^ Clients in BAME (n = 14), physical disability (n = 7), and high deprivation (n = 15) sub-groups all noted access to the online IHLS sessions to be a key barrier to continued IHLS session attendance during the COVID-19 lockdown. IHLS practitioners (n = 8) also noted barriers to delivery due to a lack of access to the correct equipment.

An increased understanding as to how people use their digital devices is warranted, from sharing laptops and mobile phones with family, housemates, and friends to ‘renting them for a day’ to accessing funds to meet basic needs. If we are not sure who is accessing the device, then session privacy and subsequent research validity is questioned.^17^ For online health behaviour change programmes to impact those who are most vulnerable, we must be vigilant when addressing these disparities.

#### Rapport

Digital technologies and smartphone apps also present a novel platform for the remote delivery of lifestyle interventions.^18^ However, further research is needed to establish how this can be done in an engaging and effective way, to reach those individuals and communities who are most deprived and vulnerable. Clients within manual worker (n = 5) and high-deprivation (n = 12) sub-groups noted feeling ‘disconnected’ from the practitioners and online sessions due to a lack of prior experience with technology. However, both IHLS practitioners (n = 5) and client attendees across all sub-groups (n = 14) were happy with the practitioner–client interactions and where applicable, client–client rapport was also built when individuals were able to access the online sessions.

#### Support

Previous programmes utilizing online health behaviour change offers to deliver remote lifestyle interventions have encountered issues of real-world engagement^19^ and bias.^17^ This can largely be put down to insufficient levels of practitioner and client guidance and support in how best to engage with such offers.^20^ Ensuring that plans for encouraging and maintaining meaningful engagement are in place before rolling out such programmes or services is vital. Workflow integration issues are also critical to consider, and the lack of attention here can lead to low uptake and support by both clients and practitioners.^21^ In contrast to such findings, clients from the WM and smoking cessation sessions reported favourable results (n = 58) in relation to the options of support provided when delivering, accessing, and engaging with the online sessions. These included telephone, email, and social media (e.g. Facebook, Instagram, and Twitter) session reminders as well as online resources on how to access and engage with sessions provided through the website.

However, CYP attending WM sessions, and adult clients attending PA sessions reported that there was a lack of support and contact (n = 7) which left them feeling ‘unwanted’. Such findings show that although there is potential for online health behaviour change offers to make a difference among under-served and vulnerable populations, further research remains necessary to explore how best to ensure such offers are scalable and accessible to all.

#### Perceived competence

In support of equity and social justice, a critical part of reducing digital attrition is ensuring all practitioners, clients, and carers, especially those who are most deprived and vulnerable, have the digital literacy and competency to partake in online offers.^17^ Negative comments were noted among clients within BAME (n = 16), manual worker (n = 4), and high deprivation (n = 13) sub-groups regarding competence to engage with the digital offers. A client from the BAME sub-group noted:

Frameworks for competencies already exist^22^ and should be implemented in the initial design of any digital health platform, although such training was not possible, given the speed at which the current pandemic influenced service provision. There is clearly merit in supporting increased access to online health behaviour change offers into the future.^23^ Further research may be valuable in exploring how competence support could be strengthened prior to and during digital platform use and whether the most effective techniques to achieve this differ between both population (e.g. CYP, adults, and older adults) and intervention objectives (e.g. WM and smoking cessation).

#### Session delivery and content

Current evidence for the successful implementation of online health behaviour change offers is limited.^24^ Previous research shows that the most effective offers are the ones that can be individually tailored to each client and fit with their personal behaviour change goals and needs.^17^ This is especially important among the vulnerable and under-served.^8^

Current National Health Service (NHS) England policy emphasizes a person-centred approach,^25^ which has potential for early detection of health decline and adjustments to be made. The IHLS provider adopts such an approach and this was reflected in the positive comments across all client sub-groups (n = 50) regarding session delivery and content.

Although many clients were aware that a transition to online delivery was compulsory, concurrent with previous research,^17^ it was noted across all sub-groups that there was a preference to return to ‘normal’ face-to-face delivery as soon as possible. However, it is noteworthy that compared to traditional interpersonal interventions, there remains little evidence of the effectiveness of exclusively digital interventions to encourage a healthy diet, PA, or WM.^26^ A single form of service delivery is never likely to meet all individual’s needs. However, given the frequency of digital health interventions is increasing rapidly, more advanced methodologies are needed to explore the components that can make such interventions successful for as many individuals as possible. Among CYP, technology, and in particular social media, has been found to be fundamental to overcome the stress and strain of lockdown. The use of social media tools have been reported to facilitate self-esteem, identity exploration, aspirational development, and provide CYP an opportunity to explore knowledge and establish new friendships.^27^ During lockdown, the average time spent on technology among CYP was more than 6 h a day for educational purposes and 4–6 h a day for recreational activities.^28^ Despite this, CYP participation in the outlined IHLS was relatively low. Hence, future research should explore engagement approaches and techniques to reach as significant a proportion of eligible CYP as possible. Consequently, process evaluations of implementation fidelity should become an integral part of the delivery and evaluation of all digital health behaviour change research. Incorporating both quantitative (e.g. frequency counts of number of session items delivered) and qualitative (e.g. interviews) measures of implementation fidelity through comprehensive frameworks can allow future researchers to accurately measure engagement delivery and session impact.

A significant strength of this study is that we assessed the COVID-19 lockdown impact among vulnerable and under-served groups, where there is a disproportionate effect of COVID-19,^29^ across WM, smoking cessation, and PA health behaviour interventions, also key additional risk factors for COVID-19 outcomes.^29^ Methodological strengths include the analysis process which provides an evidence-based foundation for the development and implementation of future interventions. Consistency of themes, data credibility, transferability, and dependability were achieved through the process of inter-rater reliability.^16^ Finally, to ensure completeness, the article was prepared in line with the 21-point checklist outlined in the standards for reporting qualitative research (SRQR).^30^ Study limitations are also noted. A small convenience sub-sample of clients from the target attrition groups were recruited via convenience sampling methods. This, along with a lack of representation of non-attenders, means that the results cannot be considered generalisable. The subjective nature of the data are also a limitation, as is the presence of self-selection bias which resulted from the convenience sampling methods adopted. Given health behaviour change intervention attrition rates vary considerably, from 10% to more than 80%, depending on the type and setting of the treatment programme,^7^ future research should seek to identify barriers, and facilitators among larger sample sizes of clients not currently/planning to partake in a health behaviour change intervention.

## Conclusion

While recognizing that there were challenges in the speed of transition from face-to-face to online delivery, which was necessary for IHLS continuation, the largely positive comments throughout have highlighted the applicability of online services during periods of lockdown, as well as the capabilities of the IHLS practitioners in maintaining delivery of effective, individually tailored sessions to the majority, regardless of client age, gender and background. Learning from decades of prior research and experience, a single form of service delivery is never likely to meet all individual’s needs. Hence, hybrid solutions that offer a blend of face-to-face and online or app-based treatment tailored as best as they can be to individual needs may be the most effective solution beyond the current pandemic to ensure competency for future lockdown situations among as significant a proportion of the population as possible. A focus on equity and ethics is warranted to ensure digital health truly increases access to impactful health behaviour change interventions delivered through online services. The COVID-19 pandemic may be the defining moment for online health behaviour change offers, given the frequency of use of such interventions during lockdown. Ensuring the right technological resources, training and support are present for the delivery of digital health offers is paramount to encourage adoption across as many individuals as possible.

## Supplemental Material

sj-docx-1-smo-10.1177_20503121211054362 – Supplemental material for Exploring reasons for attrition among vulnerable and under-served sub-groups across an online integrated healthy lifestyles service during COVID-19 Click here for additional data file. Supplemental material, sj-docx-1-smo-10.1177_20503121211054362 for Exploring reasons for attrition among vulnerable and under-served sub-groups across an online integrated healthy lifestyles service during COVID-19 by George J Sanders, Carlton Cooke and Paul Gately in SAGE Open Medicine
