# Supplementary material for: Exploring reasons for attrition among vulnerable and under-served sub-groups across an online integrated healthy lifestyles service during COVID-19
Source: SAGE Open Med. 2021 Oct 22;9:20503121211054362. doi: 10.1177/20503121211054362 (PMC8543558; doi:10.1177/20503121211054362)
Supplement: sj-docx-1-smo-10.1177_20503121211054362 – Supplemental material for Exploring reasons for attrition among vulnerable and under-served sub-groups across an online integrated healthy lifestyles service during COVID-19 [file sj-docx-1-smo-10.1177_20503121211054362.docx]

**INTEGRATED HEALTHY LIFESTYLE SERVICE ATTRITION DURING COVID-19 –**

**Example Interview Questions**

*Questions…*

1. Could you provide a brief description of the IHLS service/s you are/ have been involved with during COVID-19?
2. How long have you been attending IHLS sessions?
3. Which aspects of the sessions are enjoying the most? (e.g. delivery, session content, engaging with other participants).
4. What are the benefits of the sessions?
5. Have there been any challenges (expected or unexpected) with your continued attendance at IHLS service sessions throughout the COVID-19 pandemic? If yes, what were these & how have/ are these being overcome?
6. What has been done well throughout the COVID-19 pandemic within the IHLS service sessions you have attended (e.g. novel delivery techniques, increased engagement from certain groups, improved outcomes etc).
7. During COVID-19, do you feel that IHLS staff have been sufficiently trained in how to lead virtual sessions? If no, please explain what can be done better.
8. Has X (the county-wide IHLS) supported you to keep attending sessions throughout COVID-19? If yes, how? (e.g. virtual sessions, weekly update emails, at home plans etc.) If no, what could have been done better?
9. Are there any individuals who have positively influenced your continued attendance at OneLife Suffolk service sessions during COVID-19? If yes, who? (e.g. service practitioners, family members, friends, high profile models etc.)
10. How would you rate the delivery of the IHLS service sessions out of 10 during COVID-19? (e.g., rapport, engagement and clarity of delivery.) Please explain.
11. Is there progression of content difficulty throughout the weeks? Specific examples?
12. Do you feel that the session content delivered is appropriate for your abilities? If no, please explain what could be done better.
13. Is there anything you think could be improved upon in future sessions? (e.g., new content, equipment, facilities, timing, support networks).
14. What can be done to recruit more participants onto the IHLS sessions during COVID-19?
15. Are you aware of any of the other IHLS services? (e.g., weight management, physical activity, smoking cessation, health walk and NHS health check interventions).
16. How likely are you to recommend IHLS services to potential service users given your experience during the COVID-19 pandemic?
17. Is there anything you wish to add/feedback?
